# A controlled evaluation of social prescribing on loneliness for adults in Queensland: 8-week outcomes

**DOI:** 10.3389/fpsyg.2024.1359855

**Published:** 2024-04-12

**Authors:** Genevieve A. Dingle, Leah S. Sharman, Shaun Hayes, Catherine Haslam, Tegan Cruwys, Jolanda Jetten, S. Alexander Haslam, Niamh McNamara, David Chua, James R. Baker, Tracey Johnson

**Affiliations:** ^1^School of Psychology, The University of Queensland, Brisbane, QLD, Australia; ^2^Research School of Psychology, Australian National University, Canberra, ACT, Australia; ^3^School of Social Sciences, Nottingham Trent University, Nottingham, United Kingdom; ^4^Inala Primary Care, Brisbane, QLD, Australia; ^5^Centre for Community Health and Wellbeing, The University of Queensland, Brisbane, QLD, Australia; ^6^Primary and Community Care Services, Gold Coast, QLD, Australia; ^7^Faculty of Health, Southern Cross University, Lismore, NSW, Australia

**Keywords:** loneliness, social prescribing, group programs, community, health

## Abstract

**Introduction:**

There have been few controlled evaluations of Social Prescribing (SP), in which link workers support lonely individuals to engage with community-based social activities. This study reports early outcomes of a trial comparing General Practitioner treatment-as-usual (TAU) with TAU combined with Social Prescribing (SP) in adults experiencing loneliness in Queensland.

**Methods:**

Participants were 114 individuals who were non-randomly assigned to one of two conditions (SP, *n* = 63; TAU, *n* = 51) and assessed at baseline and 8 weeks, on primary outcomes (loneliness, well-being, health service use in past 2 months) and secondary outcomes (social anxiety, psychological distress, social trust).

**Results:**

Retention was high (79.4%) in the SP condition. Time × condition interaction effects were found for loneliness and social trust, with improvement observed only in SP participants over the 8-week period. SP participants reported significant improvement on all other outcomes with small-to-moderate effect sizes (ULS-8 loneliness, wellbeing, psychological distress, social anxiety). However, interaction effects did not reach significance.

**Discussion:**

Social prescribing effects were small to moderate at the 8-week follow up. Group-based activities are available in communities across Australia, however, further research using well-matched control samples and longer-term follow ups are required to provide robust evidence to support a wider roll out.

## 1 Introduction

An estimated one in three adults in Australia (Baker, 2012) and Aotearoa New Zealand (Hawkins-Elder et al., 2018) experience significant loneliness; an aversive experience related to a perceived discrepancy between desired and actual social connection. Although loneliness affects people from all backgrounds, it is more common among young adults and older adults, people who live alone (particularly single parents), people who are unemployed, people from an ethnic or sexual minority group, and those living with a disability or chronic disease (Mental Health Foundation (UK), 2022). In the wake of COVID-19 lockdowns and physical distancing policies designed to physically isolate individuals, there has been increased recognition of loneliness as a serious public health concern (Pai and Vella, 2021). In part, this is due to bi-directional relationships between loneliness and mental disorders such as depression (Killgore et al., 2020), social anxiety (Lim et al., 2016), psychoses (Nevarez-Flores et al., 2022), substance use disorders (Ingram et al., 2020), and a range of chronic diseases (Park et al., 2020). Loneliness is also associated with lower health-related quality of life and increased health care utilization, including frequent attendance at General Practitioners (GP) and hospital emergency departments adding burden to the health system and economy (Cruwys et al., 2018; Mosen et al., 2021). Inversely, a meta-analysis of 148 studies revealed that social support and social integration were key factors for reduced mortality risk (Holt-Lunstad et al., 2010). There is therefore an urgent need for accessible and effective solutions to loneliness in the community.

Social Prescribing (SP) is a novel and promising solution to loneliness that has been widely implemented in the United Kingdom (Global Social Prescribing Alliance, 2022). There are numerous models of SP, including general practice-based and community-based schemes (Dingle and Sharman, 2022). The most comprehensive model has three steps: (1) the person is referred to SP, usually by a GP (2) the person consults with a link worker to assess their interests and barriers to social connection; and (3) the person is supported to engage in social activities available within their local community. In Australia, SP programs are fewer and more diverse than in the UK, with programs resourced through a range of national, state, and private funding schemes and operating in GP clinics, community centers and other organizations such as workplace injury insurance (Aggar et al., 2020). Link workers have diverse professional backgrounds. In Australia, they are typically upskilled health professionals (e.g., nurses, social workers) who have extensive local knowledge of the social group programs and services in their community and use a range of interpersonal, community development and health promotion skills in their roles (Sharman et al., 2022). International evidence supports the efficacy of SP in various settings and populations, with benefits for wellbeing, quality of life, patient activation, health-related confidence, community involvement and experience of services, reduced anxiety, emotional problems, loneliness, and healthcare use (Chatterjee et al., 2018; Kellezi et al., 2019). Yet, despite promising preliminary evidence, there have been few controlled evaluations of SP, and this has led some to criticize its rapid roll out in the UK (Husk et al., 2019).

The current study was designed to address this limitation by providing a registered controlled study of SP in lonely community-dwelling adults (Dingle et al., 2022). A randomized controlled design was not considered suitable for several pragmatic reasons including that the study was conducted across several sites, with different models of social prescribing, and an uncertain recruitment rate within each site. These challenges made it unsuitable to conduct a randomized controlled design (in which cases there is typically a consistent flow of referrals in a single health service that can be randomized at the group level to one or other condition). Further, it is difficult to randomize lonely people to social group programs if they have no interest in attending them. Finally, it would be ethically questionable to withhold social group programs from lonely people who are keen to engage in them. Instead, given the research showing that lonely people are likely to attend their GP more frequently (Cruwys et al., 2018), we used a parallel controlled design to evaluate outcomes for individuals who were referred to SP in either a GP-based or community center-based model, and compared them with frequent attending patients receiving treatment as usual (TAU) from GP clinics in the same locations as the Social Prescribing programs. This paper reports on participants’ retention and engagement in SP, and the early (8-week) outcomes of the study. A companion paper will report on a test of the Social Identity-informed theoretical model that was applied with the aim of understanding the mechanisms through which SP might work^1^.

Compared to TAU, SP participants were expected to show greater improvement over time in loneliness and wellbeing, and a shift away from the use of health services (GP and hospital services) toward social and community services where their social needs might be more effectively met. Compared to TAU, SP participants were also expected to show secondary improvements in secondary —social anxiety, psychological distress, and social trust - which commonly co-occur with loneliness and have been found to act as barriers (social anxiety and distress) or facilitators (social trust) to people’s engagement with social group programs (Sharman et al., 2022).

## 2 Materials and methods

### 2.1 Design and participants

**Figure 1:**
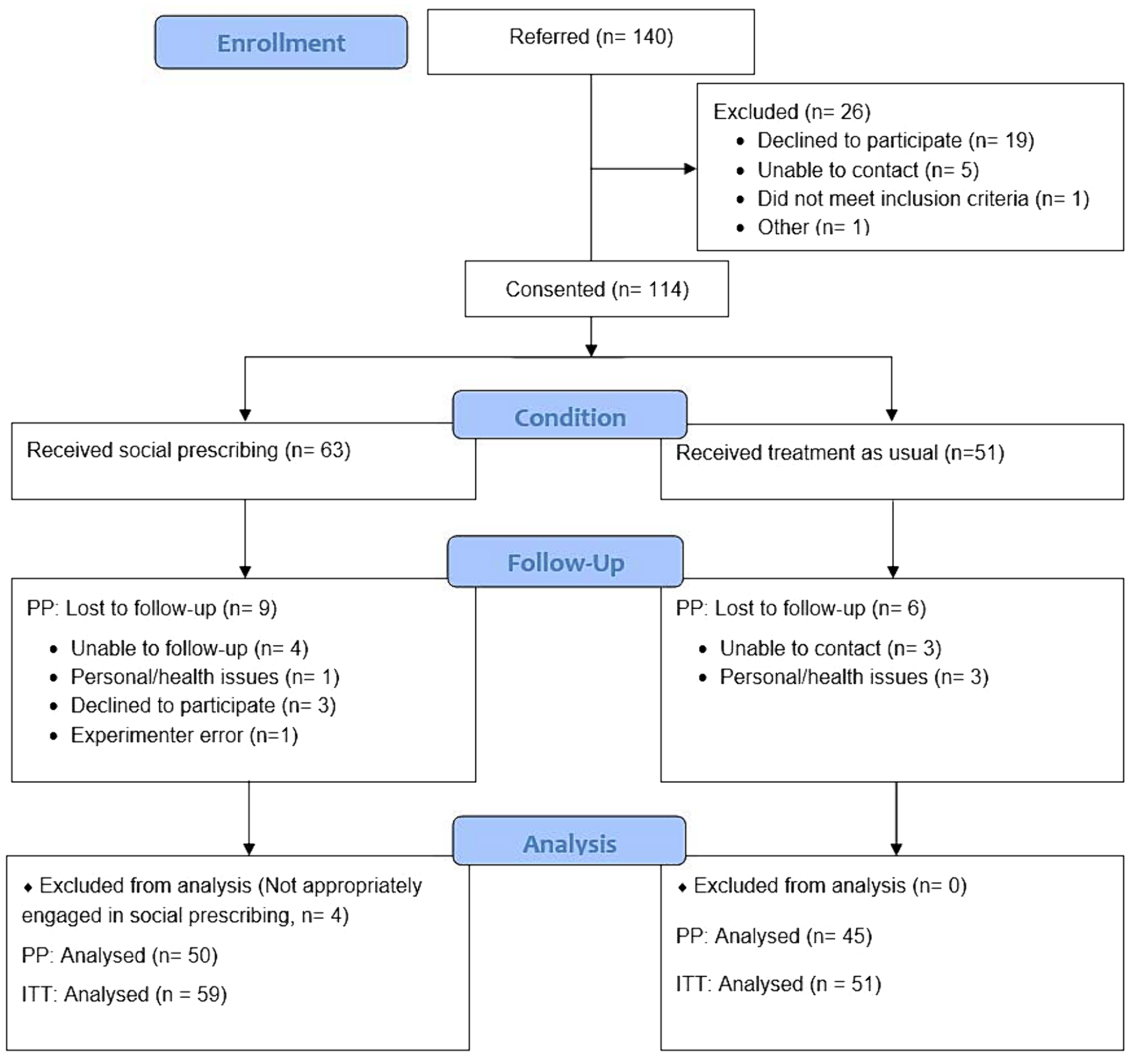
CONSORT diagram for the controlled parallel design study evaluating Social Prescribing vs. GP Treatment As Usual for lonely community-dwelling adults.

The study followed a 2 (condition: SP or TAU) × 2 (timepoint: baseline, 8 weeks later) non-randomized controlled design. Participants were 114 community dwelling adults experiencing loneliness based on self-report and/or identified by their health or social care workers. They were recruited from one of five sites in Southeast Queensland in areas with diverse populations in terms of socioeconomic and cultural backgrounds: a community center in a southern suburb of Brisbane (SP), a GP clinic in the same suburb (TAU), a GP clinic in a southwestern suburb of Brisbane (SP and TAU), a community center in a northern suburb of Brisbane (SP), and a combined GP and community centre in a regional city located about an hour’s drive south of Brisbane (SP). Of the SP participants, 20 referrals came from medical services (hospital or GP), 20 from community services and 13 were self or family referrals (6 missing data). The flow of participants through the study is shown in the CONSORT diagram in Figure 1. Researchers developed rapport with participants in the first assessment and so it was not possible to conduct blinded follow-up assessments from the participants’ condition. An *a priori* power analysis indicated that a sample size of 90 would be required to detect a significant difference between groups with a medium effect size (*f* = 0.30) on primary outcome variables, with a power of 0.8 and a default alpha of 0.05, using the planned 2 × 2 mixed analysis of variance. Our goal for recruitment was therefore to continue until we reached 90 with data at both time points (i.e., 45 in each condition).

Social Prescribing (SP) participants were 63 adults experiencing loneliness who were referred to SP programs. They were an average age of 54.67 years (SD = 15.31), the majority identified as female (72.9%), and most were not in a married /cohabiting relationship (see Table 1 for details). None were Aboriginal and/or Torres Strait Islander. However, there was some cultural diversity in that 19% spoke a first language other than English. Many had completed a post-secondary school qualification, but most were not in full-time employment. Exclusion criteria were that the individual reported acute symptoms (e.g., suicidal ideation, manic or agitated behavior) or an acute social issue that would interfere with their capacity to engage with SP. In such cases, the link worker referred them on to a more suitable service.

**Table 1:** Demographic characteristics of the participants in the Social Prescribing (*n* = 63) and GP Treatment-as-Usual (*n* = 51) conditions.

Measure	Social Prescribing	GP Treatment-as-Usual	Independent groups *t*-test
	Mean (SD) / Frequency	Mean (SD) / Frequency	
Age	54.67 (15.31)	64.92 (16.02)	*t*(106) = 3.394, *p* < 0.001
Days paid work past month	4.87 (9.20)	7.75 (9.75)	*t*(21) = 0.701, *p* = 0.245
Days volunteer work past month	3.06 (3.55)	3.75 (6.09)	*t*(22) = 0.352, *p* = 0.364
Number of dependents	0.66 (1.07)	0.62 (1.05)	*t*(104) = −0.198, *p* = 0.422
Gender			
Female	72.9%	56.9%	*χ*^2^(110) =3.836, *p* = 0.147
Relationship status			
Single	49.2%	14%	*χ*^2^(109) = 25.324, *p* < 0.001
Married/cohabiting	13.6%	50%	
Separated/divorced	27.1%	16%	
In a relationship (not cohabiting)	1.7%	4%	
Widowed	8.5%	16%	
Education level			
Less than year 12	19%	41.2%	*χ*^2^(109) = 7.106, *p* = 0.069
Year 12	12.1%	11.8%	
Certificate/diploma	37.9%	29.4%	
Univ. degree or higher	31%	17.6%	
First language English	81%	90.2%	*χ*^2^(109) = 1.819, *p* = 0.177
Aboriginal/Torres Strait Islander	0%	8%	Fishers exact, *p* = 0.043

Treatment as Usual (TAU): 51 adults who were frequent attenders at the GP clinics - defined as 12 or more times each year over a two-year period - were contacted by telephone by the researchers and invited to participate in the study. These patients had access to referral pathways into SP programs, but either declined to be referred, referral was not feasible, or their GP did not consider their referral necessary. The TAU patients had a mean age of 64.92 years (SD = 16.02), which was significantly older than the SP sample. A majority (56.9%) were female, 8% were Aboriginal and/or Torres Strait Islander people, and 10% spoke a first language other than English. Education level and days in paid or volunteer work in the past month were not significantly different from the SP sample (Table 1).

### 2.2 Measures

#### 2.2.1 Retention and SP engagement

Retention was assessed as a simple proportion (%) of participants who consented to participate in the study and completed the baseline assessment, who were retained at the 8-week assessment in each condition. SP engagement was presented in terms of the type of community group program participants were engaging with at the 8 week follow up (i.e., arts and creative activity; physical and outdoor activity; educational courses; others).

#### 2.2.2 Loneliness

Loneliness was assessed using the 8-item UCLA Loneliness Scale (ULS-8) (Hays and DiMatteo, 1987), αT1 = 0.90, αT2 = 0.91 Responses were recorded on a 4-point scale (1 = never to 4 = often). Following the guidelines for measuring loneliness published by What Works Wellbeing in the UK (What Works Wellbeing, 2019) and Ending Loneliness Together in Australia (Ending Loneliness Together, 2021), we also administered a direct measure of loneliness; the single item ‘How often do you feel lonely?’, rated on a 5-point scale from 1 = never to 5 = often/always.

#### 2.2.3 Wellbeing

Wellbeing was measured using the 14-item Warwick Edinburgh Mental Wellbeing Scale (Tennant et al., 2006), αT1 = 0.93, αT2 = 0.94. Items asked respondents how often they had experienced various psychological states over the past 2 weeks (e.g., ‘I’ve been feeling relaxed’) on a 5-point rating scale from 1 = none of the time to 5 = all of the time. Scores were summed to produce a total score in the range from 14 to 70, with higher scores corresponding to a higher level of mental wellbeing.

#### 2.2.4 Health service use

Health Service Use was measured by the self-reported number of visits to five types of health services in the past 2 months, adapted from Kellezi et al. (2016). The list was: (1) visits to the GP (in person, over the phone, or via home visit); (2) hospital, (3) community mental health services, (4) individual therapy (counselor, psychologist, psychiatrist) and (5) social worker. A total number of health visits (past 2 months) was calculated and number of visits to each type of health service were also considered separately.

#### 2.2.5 Social anxiety

Social Anxiety was measured with the three-item social phobia inventory (mini-SPIN) (Seeley-Wait et al., 2009), (αT1 = 0.80, αT2 = 0.80) asked respondents to rate how much each item (e.g., ‘Being embarrassed or looking stupid are among my worst fears’) had bothered them in the past week on scale from 0 = never to 4 = extremely. Item scores were summed to create a total score in the range from 0 to 12, and the cut off score of 6+ is interpreted as clinically meaningful.

#### 2.2.6 Psychological distress

Psychological distress was assessed using the well-established 6-item scale by Kessler (K6) (Kessler et al., 2002), αT1 = 0.93, αT2 = 0.92, which asked respondents to indicate how often they had experienced 3 depression symptoms and 3 anxiety symptoms over the past 30 days, on a scale from 0 = never to 4 = always. A total score in the range of 0 to 24 is calculated and a score of 13+ is interpreted as clinically elevated (Furukawa et al., 2003).

#### 2.2.7 Trust

Due to a lack of validated measures of general social trust suited to our study, we used an adapted version of the Cognitive Trust in Service Relationships Scale (Johnson and Grayson, 2005) by removing the service relationship components of the items (αT1 = 0.74, αT2 = 0.76). Items such as ‘I feel I can trust others’ advice to me’ were rated from 1 = strongly disagree to 7 = strongly agree.

### 2.3 Procedure

All participants provided written informed consent, as described in the research protocol approved by the University of Queensland human research ethics committee, #2019002287. Data were collected via survey by researchers either in-person or online when in-person data collection was not feasible. Participants were reimbursed with AUD $40 vouchers at T1 and T2. Further details of the methodology are published in the protocol paper (Dingle et al., 2022) and on the ANZCTR, retrospectively registered 08/06/2022.^2^

## 3 Results

### 3.1 Descriptives and baseline differences

Descriptive statistics for all primary and secondary variables are presented in Table 2. There were significant between-group differences at baseline on loneliness, wellbeing, psychological distress, and social anxiety (Table 2, middle column), with the participants in the SP condition reporting being more vulnerable on these measures than TAU participants.

**Table 2:** Descriptive analyses on loneliness, wellbeing, psychological distress, social anxiety, and trust at baseline and 8 week follow up in the Social Prescribing and GP TAU samples.

Measure	Social prescribing baseline Mean (SD)	Treatment as usual baseline Mean (SD)	Independent groups *t*-test (Baseline; two tailed)	Social prescribing +8 Weeks Mean (SD)	Treatment as usual +8 Weeks Mean (SD)
Primary outcomes					
8-item UCLA loneliness	22.94 (4.84)	19.13 (5.57)	*t*(106) = −3.428, *p* < 0.001	21.66 (5.50)	18.6 (5.46)
Direct loneliness	3.82 (1.17)	2.98 (1.10)	*t*(105) = −4.103, *p* < 0.001	3.48 (1.18)	3.16 (1.23)
Wellbeing (WEMWB Scale)	42.46 (10.26)	47.38 (9.83)	*t*(102) = 2.70, *p* = 0.008	43.43 (11.37)	45.24 (11.61)
Secondary outcomes					
Psychological Distress (K6)	11.88 (5.98)	8.10 (5.73)	*t*(105) = −3.328, *p* = 0.001	10.73 (6.38)	8.91 (6.46)
Social Anxiety (miniSPIN)	5.93 (3.27)	4.89 (3.32)	*t*(106) = −2.115, *p* = 0.037	5.04 (2.99)	3.89 (3.10)
Social Trust	3.54 (0.96)	3.75 (1.11)	*t*(106) = 1.08, *p* = 0.283	3.86 (1.14)	3.59 (1.15)

### 3.2 Retention and engagement

Participant retention rates were high at the 8-week follow up (79.4% in the SP and 88.2% in TAU condition), which shows the acceptability of the intervention for lonely community-dwelling adults. Of the 64 social group programs that SP participants were attending at the 8-week follow-up via SP, 24 were arts and creative activities, 17 were physical and outdoor activities, 12 were educational programs, and 11 were others (such as social support and general socializing programs).

### 3.3 Primary outcomes

To assess the effect of the SP intervention, group differences in trends over time on the primary measures were examined using a series of mixed-effects repeated measures (MMRM) models, which specified timepoint, participant, and condition as levels in the analyses. MMRM is a full-information maximization likelihood estimation strategy that can model all available data even when some observations are missing, and thus honors the intention-to-treat principle (Molenberghs et al., 2004). For all hypothesis testing, per-protocol analyses were conducted with data from *n* = 50 in SP who completed both assessments and engaged with a community group activity via SP; and *n* = 45 in TAU who completed both assessments (see Table 3). Intention-to-treat analyses on data from the full original sample can be found in the Supplementary File S1. Analyses were conducted with age, relationship status, and days between time points entered as covariates, however; only age was significant as a covariate. Therefore, the main analyses are presented with and without age as a covariate (Table 3).

**Table 3:** Results of per protocol mixed-effects repeated measures (MMRM) models comparing the 50 Social Prescribing participants with the 45 GP Treatment-as-Usual participants over the 8 weeks period on primary and secondary outcomes.

		No covariates			Age covariate*		
		*β* (SE)	df	*p*	*β* (SE)	df	*p*
Direct Loneliness	Time	0.016 (0.12)	99.05	0.192	0.162 (0.123)	99.22	0.191
	Condition	0.715 (0.18)	149.14	**0.0002**	0.521 (0.184)	149.13	**0.005**
	Time*Condition	−0.447 (0.169)	98.99	0.**010**	−0.458 (0.169)	99.14	**0.008**
Indirect Loneliness (ULS-8)	Time	−0.061 (0.117)	97.2	0.604	−0.057 (0.117)	96.68	0.627
	Condition	0.582 (0.184)	143.99	**0.002**	0.318 (0.175)	148.28	0.071
	Time*Condition	−0.051 (0.162)	97.56	0.753	−0.059 (0.162)	97.09	0.714
Mental wellbeing (WEMWB)	Time	−0.195 (0.114)	92.49	0.089	−0.194 (0.114)	92.04	0.091
	Condition	−0.481 (0.191)	141.62	**0.013**	−0.294 (0.192)	141.17	0.129
	Time*Condition	0.269 (0.161)	94.43	0.097	0.271 (0.161)	94.12	0.095
Total health	Time	−0.242 (0.165)	95.56	0.146	−0.247 (0.166)	94.60	0.140
Visits (past 2)	Condition	−0.035 (0.193)	176.33	0.856	−0.214 (0.193)	177.22	0.268
(Months)	Time*Condition	0.259 (0.227)	96.36	0.257	0.251 (0.228)	95.45	0.273
Psychological distress (K6)	Time	0.128 (0.101)	96.34	0.206	0.124 (0.101)	94.75	0.224
	Condition	0.579 (0.186)	135.18	**0.002**	0.298 (0.183)	135.45	0.105
	Time*Condition	−0.269 (0.140)	96.86	0.057	−0.270 (0.14)	95.60	0.059
Social anxiety (mini-SPIN)	Time	−0.200 (0.130)	97.35	0.128	−0.200 (0.132)	94.26	0.132
	Condition	0.429 (0.188)	152.45	**0.033**	0.247 (0.192)	148.66	0.201
	Time*Condition	−0.064 (0.179)	97.43	0.744	−0.066 (0.182)	94.68	0.717
Social Trust	Time	−0.126 (0.155)	101.84	0.399	−0.126 (0.155)	102.02	0.418
	Condition	−0.185 (0.193)	171.56	0.438	−0.241 (0.201)	167.21	0.233
	Time*Condition	−0.434 (0.214)	102.36	**0.046**	0.432 (0.214)	102.53	**0.047**

* Participant age and relationship status were significantly different between conditions (see Table 1) so these variables and the number of days between T1 and T2 were entered into analyses as potential covariates. However, only age was significant, so the results are presented here with and without age covaried. Significant results in bold type.

#### 3.3.1 Loneliness

**Figure 2:**
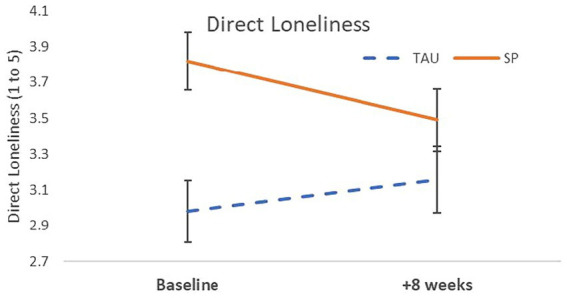
Loneliness decreased over the first 8 weeks in participants engaged in Social Prescribing but increased over time in the GP Treatment-as-Usual participants.

The direct measure of loneliness showed a significant Time × Condition interaction, *β* = −0.45, *p* = 0.01; and a main effect of Condition, *β* = 0.72, *p* < 0.001; with and without age covaried (Table 3 and Figure 2). Over the first 8 weeks, loneliness decreased in SP participants (*SE* = 0.15, *d* = −1.24) but increased in TAU (*SE* = 0.15, *d* = 0.46). The ULS-8 loneliness measure showed a significant main effect of Condition, *β* = 0.58, *p* = 0.002 (which was not significant after controlling for age; see Table 3). Follow-up analyses found that this measure of loneliness was higher both at baseline, *t*(146.8) = 3.15, *p* = 0.008, and + 8 weeks, *t*(158.7) = 1.77, *p* = 0.023, in the SP sample than in the TAU condition. Over time, there was a decrease in loneliness for both SP (*SE* = 0.64*, d* = −0.20), and for TAU (SE = 0.67, *d* = −0.12). Although these values changed in the expected directions, the interaction effect was not significant: *β* = −0.051, *p* = 0.753 (Tables 2, 3).

#### 3.3.2 Mental wellbeing

There was a significant main effect of Condition (*β* = −0.48, *p* = 0.013) indicating that SP participants’ wellbeing was significantly lower than TAU participants’ throughout the 8-week period. Changes over time were in the expected direction, with an average increase in wellbeing for SP (SE = 1.25, *d* = 0.14), and a decrease for TAU (SE = 1.25, *d* = −0.36). However, the interaction effect failed to reach significance (*β* = 0.269, *p* = 0.097), see Table 3.

#### 3.3.3 Health service use

No significant interaction was found for the total number of health visits over the past 2 months (*β* = 0.259, *p* = 0.257), see Table 3. However, effects emerged when number of visits to specific types of health services were analyzed (see Table 4) and figures in Supplementary File S2. Visits to a GP showed a significant effect of condition (*p* < 0.001), such that TAU reported higher attendance than SP at both time points. Hospital care showed a main effect of Time (*p* = 0.031), decreasing in both conditions. Use of community mental health services and individual therapy services (i.e., Psychologist, Counselor, or Psychiatrist) showed significant effects of Condition with higher attendance rates for SP than for TAU at both time points (*p* = 0.003 and *p* = 0.018, respectively). No significant effects were found for visits to social workers.

**Table 4:** Health service use in the past 2 months reported by participants in the Social Prescribing and the GP Treatment-as-Usual condition.

	Social Prescribing		Treatment as Usual		*t*-test
	T1 Mean (SD)	T2 Mean (SD)	T1 Mean (SD)	T2 Mean (SD)	W = within (time), B = between (condition), t*C = time×condition interaction. Sig effects in bold.
General Practitioner	1.44 (1.59)	1.66 (1.36)	2.97 (1.80)	2.45 (2.34)	*W*: *F*(1.89) = 0.48, *p* = 0.489, *η*^2^ = 0.005
					***B: F*(1.92) = 14.15, *p* < 0.001, *η***^**2**^ **= 0.137**
					t*C: F(1.89) = 2.92, *p* = 0.091, *η*^2^ = 0.032
Hospital inpatient care	0.33 (0.64)	0.27 (0.73)	0.67 (1.04)	0.31 (0.66)	***W: F*(1.91) = 4.81, *p* = 0.031, *η***^**2**^ **= 0.050**
					*B: F*(1.91) = 2.13, *p* = 0.148, η^2^ = 0.023
					t*C: F(1.92) = 2.26, *p* = 0.136, *η*^2^ = 0.024
Community mental health services	1.03 (2.03)	0.83 (1.65)	0.08 (0.33)	0.35 (1.27)	*W: F*(1.92) = 0.034, *p* = 0.854, *η*^2^ = 0.000
					***B: F*(1.90) = 9.05, *p* = 0.003, *η***^**2**^ **= 0.090**
					t*C: F(1.92) = 1.45, *p* = 0.232, η^2^ = 0.015
Individual therapy	1.36 (1.83)	1.44 (2.48)	0.70 (1.80)	0.39 (2.48)	*W: F*(1.90) = 0.49, *p* = 0.486, *η*^2^ = 0.005
					***B: F*(1.90) = 5.78, *p* = 0.018, *η***^**2**^ **= 0.060**
					t*C: *F*(1.90) = 1.31, *p* = 0.225, *η*^2^ = 0.014
Social worker	0.59 (1.46)	0.58 (1.26)	0.81 (3.79)	0.10 (0.39)	*W: F*(1.90) = 1.73, *p* = 0.192, *η*^2^ = 0.019
					*B: F*(1.90) = 0.14, *p* = 0.710, *η*^2^ = 0.002
					t*C: F(1.90) = 1.66, *p* = 0.200, *η*^2^ = 0.018

Significant findings in bold type.

### 3.4 Secondary outcomes

#### 3.4.1 Psychological distress

The main effect of Condition was significant for psychological distress (K6), *β* = −0.58, *p* = 0.002. Follow-up analyses revealed a significant difference between conditions at baseline, *t*(135.5) = 2.87, *p* = 0.017, with the SP group significantly more distressed than TAU. The SP condition reported an average decrease in distress over the 8-weeks (SE = 0.63, *d* = −0.29) while the TAU condition reported an average increase (SE = 0.64, *d* = 0.26). However, the Time × Condition interaction failed to reach significance (*p* = 0.057), see Table 3.

#### 3.4.2 Social anxiety

Social anxiety showed a main effect of Condition, *β* = 0.43, *p* = 0.033, but no Time × Condition interaction and no main effect of Time. This corresponded to an average decrease in social anxiety for both SP (SE = 0.41, *d* = −0.45) and TAU (SE = 0.43, *d* = −0.30), see Table 3.

#### 3.4.3 Social trust

**Figure 3:**
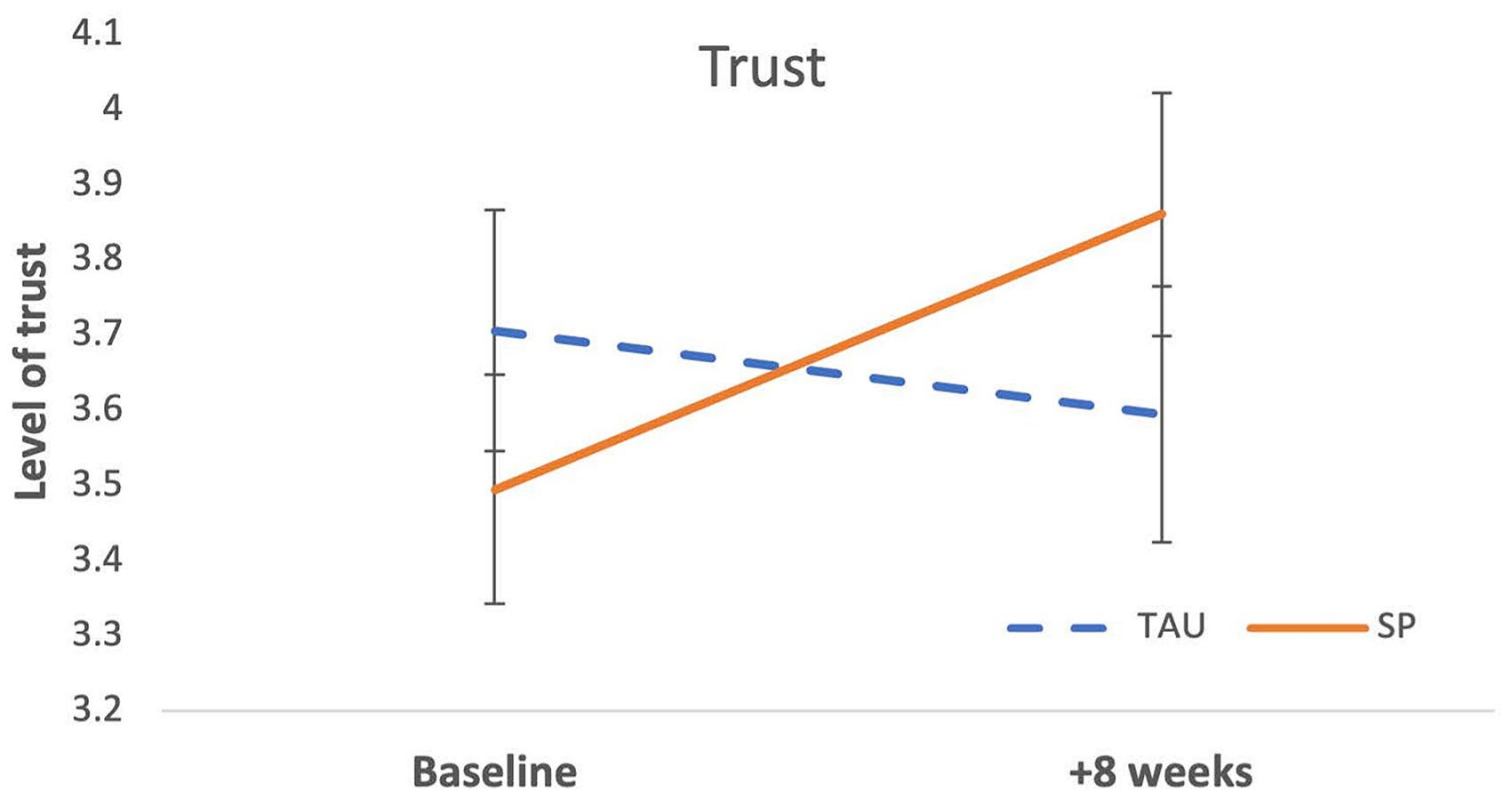
Trust increased over 8 weeks in the Social Prescribing participants and decreased over time in the participants receiving GP Treatment-as-Usual.

A significant Time × Condition interaction was revealed for trust, *β* = −0.43, *p* = 0.046, see Table 3. Trust increased among SP participants over time (SE = 0.17, *d* = 0.41), but decreased in TAU (SE = 0.16, *d* = −0.17), see Table 3 and Figure 3.

## 4 Discussion

This study is one of the first controlled evaluations of an SP intervention for loneliness in a heterogenous sample of lonely community-dwelling adults. After an 8-week period there were changes in the expected direction on all primary and secondary measures in the SP participants. When considered in comparison to the GP TAU condition, there were significant interaction effects on the outcomes of loneliness and trust, indicating that people who engaged in SP felt less lonely and more trusting of others after 8 weeks while people receiving GP TAU did not report these benefits. The interaction effect on psychological distress (depression and anxiety symptoms) did not reach conventional significance levels, but the effect size (*d* = −0.29) showed a small to medium improvement in SP participants whereas the TAU group had a small to medium deterioration (*d* = 0.26). In addition to the findings for loneliness and trust, there were main effects of condition on the ULS-8 loneliness, wellbeing, psychological distress, social anxiety, and the number of visits to the GP, community mental health services and individual therapy services (past 2 months). When scores on these measured variables were considered alongside the participants’ demographic characteristics, it appears that the two conditions were not well matched. This is a limitation of the non-randomized design, which left the project vulnerable to selection bias in the participants recruited to the two conditions. Although all participants were community-dwelling adults in the geographical locations where SP programs were offered, these data suggest that SP participants tended to experience loneliness in the context of ongoing mental health problems (i.e., higher baseline scores psychological distress, social anxiety, lower scores for wellbeing, higher number of visits to mental health and individual therapy services in the past 2 months) for while the TAU participants appear to have experienced loneliness in the context of chronic diseases (i.e., higher frequency of visits to the GP in the past 2 months). As has been noted by other researchers such as Husk et al. (2019) and colleagues, this speaks to the importance of conducting controlled evaluations and the challenge of implementing them effectively. Instead of the parallel controlled design we used in the study, an asymmetrical stepped wedge design - with a shorter control period while link workers establish the relationship and a longer intervention period - might be a better approach for future studies.

The few significant interaction effects found at 8-weeks follow up could be interpreted in two ways. One interpretation is that social prescribing is not robust in addressing loneliness and wellbeing among community dwelling adults. An alternative interpretation is that many of the effects of social prescribing take longer than 8 weeks to emerge. This begs the question: how long does it take for people experiencing chronic loneliness to show change in response to an intervention? Effects may be stronger after a longer period of engaging in social group programs, as found in previous research evaluating mental health outcomes from community groups (Dingle et al., 2021). We have conducted an 18-month follow up and will be able to report longer-term effects in due course. Other research evaluating tailored loneliness interventions have produced effects on the 8-item UCLA (Hays and DiMatteo, 1987) and other measures of loneliness over intervals of 8-weeks or less, such as GROUPS 4 HEALTH (Haslam et al., 2019), internet delivered cognitive behavior therapy (Käll et al., 2020), and a smartphone-based mindfulness training for reducing loneliness and increasing social contact (Lindsay et al., 2019). It is possible that social prescribing to community programs may take longer to produce effects on loneliness and wellbeing than tailored loneliness interventions; however, to our knowledge, no study has yet compared these approaches.

### 4.1 Limitations and challenges

There were several challenges to the study which could not have been predicted at commencement. The first was the onset of the COVID-19 pandemic which led to the closure of community-based group programs for most of the first year of the study, and ongoing disruptions in Year 2 as new virus waves emerged. During these times, link workers and other stakeholders were redeployed to emergency food relief and other services and the SP programs were paused. The second challenge was that, as noted above, the TAU condition was not equivalent to the SP condition in several respects. This is a risk of the non-randomized design which was not apparent until data collection and analysis was completed. Furthermore, it is intriguing that the results for the single-item direct measure of loneliness were significant while the results for the 8-item UCLA loneliness scale did not reach significance. Our results show that while the single item of loneliness was sensitive to change over the 8-week period, scores on the 8-item measure may not change in response to a SP intervention or may take longer for changes to occur. The single item measure asks how often respondents feel lonely (i.e., tapping into the emotional aspect of loneliness) while the 8-item UCLA scale refers to social inclusion aspects of loneliness (e.g., lacking companionship, feeling left out, and socially isolated). It is possible then, that these differences might reflect sensitivity in picking up change in these different components of loneliness. Clearly, this is speculation, but it possibility raises questions for future research about the importance of exploring different aspects of loneliness to determine their differential responsiveness to intervention. In this, our results are comparable to an earlier single-arm study of SP involving 12 adults experiencing chronic mental health problems (Aggar et al., 2021). Aggar and colleagues’ study included a six-month follow up, at which time participants showed significant improvements in quality of life and perceived health, while their scores on UCLA loneliness and K10 psychological distress did not show significant change. Our findings underscore the importance of including a direct measure of loneliness in any SP evaluation, consistent with recommendations (What Works Wellbeing, 2019; Ending Loneliness Together, 2021).

### 4.2 Conclusions and implications

The results provide some early indications that Social Prescribing is acceptable to participants and can be an effective way to help people address loneliness and develop trusted social connections in the community. Due to the number of effects that failed to reach statistical significance at 8-weeks follow up however, we can only make modest claims at this point. The implication of this for social prescribing programs is that they should not be viewed as a single intervention, but as a set of relationships and intervention components that may together help the individual address social and non-medical needs more effectively over time. As health systems respond to the COVID-19 pandemic and its aftermath, it is more important than ever to develop approaches that are accessible and strengthen participants’ connection to their communities. There have been international calls for novel approaches to protect mental wellbeing, based on theoretically informed components and the harnessing of social resources that enhance resilience in the face of social disruption exacerbated by COVID-19 (Holmes et al., 2022). SP has potential to meet this need. GPs and allied health professionals in Australia are keen to use SP, and State and Federal governments are considering SP among options for much-needed health reform (Royal Australian College of General Practitioners and Consumers Health Forum of Australia, 2020; Community Support and Services Committee, 2021; Commonwealth of Australia Department of Health, 2022; Dingle and Groarke, 2022). Future research with robust designs and longer-term follow ups are required to provide the evidence required to capitalize on this enthusiasm.

## Data availability statement

The raw data supporting the conclusions of this article will be made available by the authors, without undue reservation.

## Ethics statement

The studies involving humans were approved by University of Queensland Human Research Ethics Committee. The studies were conducted in accordance with the local legislation and institutional requirements. The participants provided their written informed consent to participate in this study.

## Author contributions

GD: Conceptualization, Formal analysis, Funding acquisition, Investigation, Methodology, Project administration, Supervision, Writing – original draft. LS: Data curation, Investigation, Methodology, Project administration, Writing – review & editing. SH: Data curation, Project administration, Writing – review & editing. CH: Conceptualization, Funding acquisition, Methodology, Writing – review & editing. TC: Conceptualization, Data curation, Funding acquisition, Methodology, Writing – review & editing. JJ: Conceptualization, Funding acquisition, Writing – review & editing. SAH: Conceptualization, Funding acquisition, Supervision, Writing – review & editing. NM: Conceptualization, Funding acquisition, Methodology, Writing – review & editing. DC: Data curation, Writing – review & editing. JB: Data curation, Investigation, Writing – review & editing. TJ: Data curation, Investigation, Writing – review & editing.

## Footnotes

^1^Hayes, S., Sharman, L. S., McNamara, N., et al. (under review). Link workers’ and clients’ perspectives on how social prescribing offers a social cure for loneliness, manuscript under review.

^2^ https://www.anzctr.org.au/ACTRN12622000801718.aspx
