# Supplementary material for: A controlled evaluation of social prescribing on loneliness for adults in Queensland: 8-week outcomes
Source: Front Psychol. 2024 Apr 12;15:1359855. doi: 10.3389/fpsyg.2024.1359855 (PMC11049426; doi:10.3389/fpsyg.2024.1359855)
Supplement: Supplementary file 1 [file Table_1.docx]

|  |  | **Per protocol** | | | **PP Age covariate** | | | |
| --- | --- | --- | --- | --- | --- | --- | --- | --- |
|  | *Predictors* | β | *CI* | *p* | β | *CI* | *p* |  |
| **Direct Loneliness** | Condition | 0.71 | 0.35 – 1.08 | **<.001** | 0.52 | 0.16 – 0.88 | **0.005** |  |
|  | Time | 0.16 | -0.08 – 0.40 | .191 | 0.16 | -0.08 – 0.40 | 0.189 |  |
|  | Interaction | -0.45 | -0.78 – -0.11 | **.009** | -0.46 | -0.79 – -0.12 | **0.007** |  |
| **Indirect Loneliness** | Condition | 0.59 | 0.22 – 0.95 | **.002** | 0.32 | -0.03 – 0.66 | 0.071 |  |
|  | Time | -0.06 | -0.29 – 0.17 | .603 | -0.06 | -0.29 – 0.17 | 0.626 |  |
|  | Interaction | -0.05 | -0.37 – 0.27 | .752 | -0.06 | -0.38 – 0.26 | 0.713 |  |
| **Mental wellbeing** | Condition | -0.48 | -0.86 – -0.10 | **.013** | -0.29 | -0.67 – 0.09 | 0.128 |  |
|  | Time | -0.2 | -0.42 – 0.03 | .087 | -0.19 | -0.42 – 0.03 | 0.09 |  |
|  | Interaction | 0.27 | -0.05 – 0.59 | .096 | 0.27 | -0.05 – 0.59 | 0.093 |  |
| **Health service usage** | Condition | 0.07 | -0.32 – 0.46 | 0.715 | -0.13 | -0.52 – 0.25 | 0.492 |  |
|  | Time | -0.44 | -0.79 – -0.10 | **0.012** | -0.47 | -0.81 – -0.12 | **0.009** |  |
|  | Interaction | 0.44 | -0.05 – 0.93 | 0.080 | 0.44 | -0.05 – 0.94 | 0.077 |  |
| **Psychological distress** | Condition | 0.54 | 0.17 – 0.91 | **.004** | 0.3 | -0.06 – 0.66 | 0.105 |  |
|  | Time | 0.13 | -0.07 – 0.33 | .217 | 0.12 | -0.08 – 0.32 | 0.222 |  |
|  | Interaction | -0.27 | -0.55 – 0.01 | .06 | -0.27 | -0.55 – 0.01 | 0.058 |  |
| **Social anxiety** | Condition | 0.45 | 0.08 – 0.83 | **.018** | 0.38 | -0.00 – 0.77 | 0.053 |  |
|  | Time | -0.18 | -0.44 – 0.07 | .153 | -0.18 | -0.44 – 0.07 | 0.157 |  |
|  | Interaction | -0.1 | -0.45 – 0.26 | .59 | -0.1 | -0.45 – 0.25 | 0.575 |  |
| **Trust** | Condition | -0.19 | -0.57 – 0.20 | .339 | -0.24 | -0.64 – 0.16 | 0.232 |  |
|  | Time | -0.13 | -0.43 – 0.18 | .42 | -0.13 | -0.43 – 0.18 | 0.417 |  |
|  | Interaction | 0.43 | 0.01 – 0.86 | **.044** | 0.43 | 0.01 – 0.85 | **0.046** |  |

Table S1.

*Results of per protocol analyses on the primary and secondary outcome variables, with age entered as a covariate (right column) and without age as a covariate (left column).*

Table S2.

*Results of Intention-to-treat analyses on the primary and secondary outcomes variables, with age entered as a covariate (right column) and without age as a covariate (left column).*

|  |  | **Intention to treat** | | | **ITT Age covariate** | | |  |
| --- | --- | --- | --- | --- | --- | --- | --- | --- |
|  | *Predictors* | β | *CI* | *p* | β | *CI* | *p* | |
| **Direct Loneliness** | Condition | 0.73 | 0.37 – 1.09 | **<0.001** | 0.52 | 0.16 – 0.88 | **0.005** | |
|  | Time | 0.16 | -0.08 – 0.40 | 0.188 | 0.16 | -0.08 – 0.40 | 0.189 | |
|  | Interaction | -0.44 | -0.77 – -0.11 | **0.009** | -0.46 | -0.79 – -0.12 | **0.007** | |
| **Indirect Loneliness** | Condition | 0.63 | 0.27 – 0.99 | **0.001** | 0.32 | -0.03 – 0.66 | 0.071 | |
|  | Time | -0.05 | -0.28 – 0.18 | 0.653 | -0.06 | -0.29 – 0.17 | 0.626 | |
|  | Interaction | -0.06 | -0.38 – 0.26 | 0.706 | -0.06 | -0.38 – 0.26 | 0.713 | |
| **Mental wellbeing** | Condition | -0.52 | -0.89 – -0.14 | **0.007** | -0.29 | -0.67 – 0.09 | 0.128 | |
|  | Time | -0.2 | -0.42 – 0.02 | 0.079 | -0.19 | -0.42 – 0.03 | 0.09 | |
|  | Interaction | 0.27 | -0.04 – 0.59 | 0.085 | 0.27 | -0.05 – 0.59 | 0.093 | |
| **Health service usage** | Condition | 0.06 | -0.32 – 0.45 | 0.755 | -0.13 | -0.52 – 0.25 | 0.492 | |
|  | Time | -0.44 | -0.78 – -0.10 | **0.013** | -0.47 | -0.81 – -0.12 | **0.009** | |
|  | Interaction | 0.45 | -0.04 – 0.93 | 0.073 | 0.44 | -0.05 – 0.94 | 0.077 | |
| **Psychological distress** | Condition | 0.58 | 0.21 – 0.95 | **0.002** | 0.3 | -0.06 – 0.66 | 0.105 | |
|  | Time | 0.13 | -0.07 – 0.33 | 0.204 | 0.12 | -0.08 – 0.32 | 0.222 | |
|  | Interaction | -0.27 | -0.55 – 0.01 | 0.056 | -0.27 | -0.55 – 0.01 | 0.058 | |
| **Social anxiety** | Condition | 0.47 | 0.10 – 0.84 | **0.013** | 0.38 | -0.00 – 0.77 | 0.053 | |
|  | Time | -0.19 | -0.44 – 0.07 | 0.147 | -0.18 | -0.44 – 0.07 | 0.157 | |
|  | Interaction | -0.1 | -0.45 – 0.25 | 0.57 | -0.1 | -0.45 – 0.25 | 0.575 | |
| **Trust** | Condition | -0.2 | -0.57 – 0.18 | 0.299 | -0.24 | -0.64 – 0.16 | 0.232 | |
|  | Time | -0.13 | -0.43 – 0.18 | 0.416 | -0.13 | -0.43 – 0.18 | 0.417 | |
|  | Interaction | 0.44 | 0.02 – 0.86 | **0.039** | 0.43 | 0.01 – 0.85 | **0.046** | |
