# Supplementary material for: A controlled evaluation of social prescribing on loneliness for adults in Queensland: 8-week outcomes
Source: Front Psychol. 2024 Apr 12;15:1359855. doi: 10.3389/fpsyg.2024.1359855 (PMC11049426; doi:10.3389/fpsyg.2024.1359855)
Supplement: Supplementary file 2 [file Presentation_1.pdf]

Supplementary file 2

Number of visits to health care in the past 2 months (total in top left panel) and by type of service (other panels) at baseline and 8-week follow up.

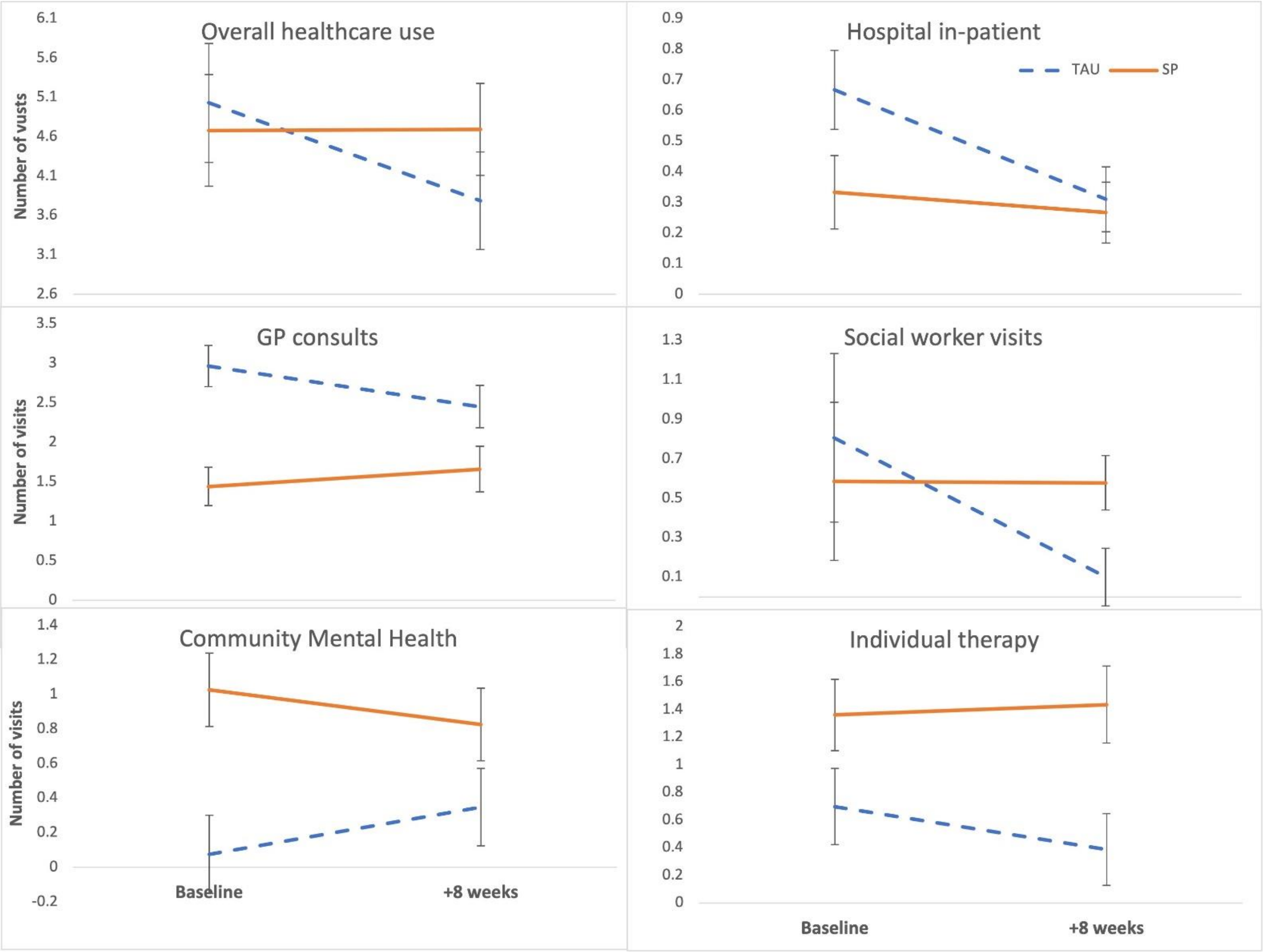

Legend: y-axis is number of visits past 2 months, with means shown for participants in the Social Prescribing condition (orange) and GP Treatment-As-Usual condition (blue); bars are standard deviations.
